# Postoperative Tobacco Cessation Improves Quality of Life, Lung Function and Long-Term Survival in Non-Small-Cell Lung Cancer Patients

**DOI:** 10.3390/cancers16020465

**Published:** 2024-01-22

**Authors:** Fabian Doerr, Tobias Leschczyk, Konstantinos Grapatsas, Hruy Menghesha, Natalie Baldes, Georg Schlachtenberger, Matthias B. Heldwein, Maximilian Michel, Alexander Quaas, Lars Hagmeyer, Katja Höpker, Thorsten Wahlers, Kaid Darwiche, Christian Taube, Martin Schuler, Khosro Hekmat, Servet Bölükbas

**Affiliations:** 1 Department of Thoracic Surgery, West German Cancer Center, University Medical Center Essen-Ruhrlandklinik, University Duisburg-Essen, 45239 Essen, Germany; 2 Department for General Surgery, St. Elisabeth Hospital Hohenlind, 50935 Cologne, Germany; 3 Department of Thoracic Surgery, Helios Clinic Bonn/Rhein-Sieg, 53123 Bonn, Germany; 4 Department of Cardiothoracic Surgery, University Hospital of Cologne, University of Cologne, 50937 Cologne, Germany; 5 Institute of Zoology, Faculty of Mathematics and Natural Sciences, University of Cologne, 50937 Cologne, Germany; 6 Institute of Pathology, University of Cologne, 50937 Cologne, Germany; 7 Clinic for Pneumology and Allergology, Bethanien Hospital GmbH Solingen, 42699 Solingen, Germany; 8 Faculty of Medicine, Clinic III for Internal Medicine, University Hospital Cologne, University of Cologne, 50937 Cologne, Germany; 9 Department of Pneumology, West German Cancer Center, University Medical Center Essen-Ruhrlandklinik, University Duisburg-Essen, 45239 Essen, Germany; 10 Department of Medical Oncology, West German Cancer Center, University Medical Center Essen, University Duisburg-Essen, 45239 Essen, Germany

**Keywords:** non-small-cell lung cancer, lobectomy, lung capacity, life quality, long-term survival, smoking cessation

## Abstract

**Simple Summary:**

This study aimed to assess the impact of postoperative smoking cessation on lung function, quality of life (QOL), and long-term survival in non-small cell lung cancer (NSCLC) patients. Two matched groups were formed including patients who quit smoking postoperatively and patients who continued smoking. One year after surgery, both groups showed a similar reduction in FEV1. However, smoking cessation was linked to improved DLCO and QOL. Importantly, patients who quit smoking postoperatively demonstrated significantly superior overall survival rates. These findings highlight the positive association between postoperative smoking cessation, enhanced QOL, and increased long-term survival in NSCLC patients, providing motivation for the implementation of smoking cessation programs.

**Abstract:**

Objectives: About 90% of all non-small cell lung cancer (NSCLC) cases are associated with inhalative tabacco smoking. Half of patients continue smoking during lung cancer therapy. We examined the effects of postoperative smoking cessation on lung function, quality of life (QOL) and long-term survival. Materials and Methods: In total, 641 patients, who underwent lobectomy between 2012 and 2019, were identified from our single institutional data base. Postoperatively, patients that actively smoked at the time of operation were offered a structured ‘smoking cessation’ program. For this retrospective analysis, two patient groups (total *n* = 90) were selected by pair matching. Group A (*n* = 60) had no postoperative tobacco smoking. Group B (*n* = 30) involved postoperative continued smoking. Lung function (FEV1, DLCO) and QOL (‘SF-36′ questionnaire) were measured 12 months postoperatively. We compared long-term outcomes using Kaplan–Meier curves. Results: The mean age in group A was 62.6 ± 12.5 years and that in group B was 64.3 ± 9.7 years (*p* = 0.82); 64% and 62%, respectively, were male (*p* = 0.46). Preoperative smoking habits were similar (‘pack years’: group A, 47 ± 31; group B, 49 ± 27; *p* = 0.87). All relevant baseline characteristics we collected were similar (*p* > 0.05). One year after lobectomy, FEV1 was reduced by 15% in both groups (*p* = 0.98). Smoking cessation was significantly associated with improved DLCO (group A: 11 ± 16%; group B: −5 ± 14%; *p* <0.001) and QOL (vitality (VT): +10 vs. −10, *p* = 0.017; physical role function (RP): +8 vs. −17, *p* = 0.012; general health perceptions (GH): +12 vs. −5, *p* = 0.024). Patients who stopped smoking postoperatively had a significantly superior overall survival (median survival: 89.8 ± 6.8 [95% CI: 76.6–103.1] months vs. 73.9 ± 3.6 [95% CI: 66.9–80.9] months, *p* = 0.034; 3-year OS rate: 96.2% vs. 81.0%, *p* = 0.02; 5-year OS rate: 80.0% vs. 64.0%, *p* = 0.016). The hazard ratio (HR) was 2.31 [95% CI: 1.04–5.13] for postoperative smoking versus tobacco cessation. Conclusion: Postoperative smoking cessation is associated with improved quality of life and lung function testing. Notably, a significant increase in long-term survival rates among non-smoking NSCLC patients was observed. These findings could serve as motivation for patients to successfully complete a non-smoking program.

## 1. Introduction

The incidence of lung cancer is expected to still rise significantly in the 2020s [1]. Not surprisingly, the leading risk factor for the development of lung cancer is the regular consumption of tobacco, which is associated with around 90 percent of all cases [2]. The loss of life expectancy is estimated to average up to 11.5 years [3]. In several European case–control studies, male active smokers were found to have a 24-fold higher risk of developing lung cancer compared to their non-smoking counterparts. In the same comparison, women show a nine-fold increased risk [4]. 

After diagnosis of lung cancer, motivation to quit smoking increases for many patients [5]. However, 20 to 40 percent manage to abstain only temporarily [2,6]. Half of the patients continue to smoke despite an increased risk of complications in lung cancer treatment [7,8]. Today, various methods for assessing functional operability and prognostic indicators for the perioperative course are known [9]. Yet, only few clear recommendations regarding smoking cessation are available. These relate in particular to the preoperative period when smoking should be stopped [9]. 

In the present study, we hypothesized that postoperative smoking cessation might improve not only quality of life and lung function but might also have a positive effect on survival. In the absence of comparable studies, we focus our investigation on encompassing three outcome measures to provide a more comprehensive and nuanced perspective on the potential benefits of quitting smoking after NSCLC surgery. The novelty of our approach might underscore the importance of exploring the synergistic effects of smoking cessation on diverse postoperative outcomes in NSCLC patients.

## 2. Materials and Methods

### 2.1. Patient Recruitment

**Figure 1:**
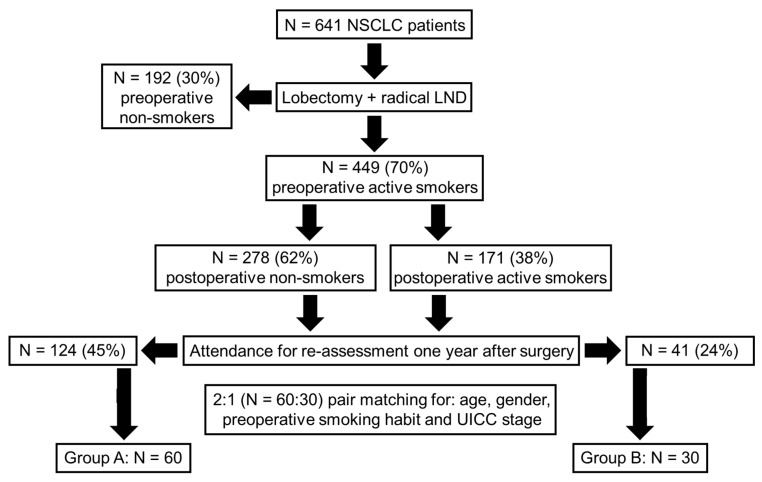
Study flow chart with inclusion and exclusion criteria. LND: lymph node dissection; UICC: union for international cancer control.

This study was registered under the number 19-1171 at the ethics committee of the University of Cologne, which waived the need for the consent of patients on 17 April 2019. Patient recruitment was between January 2012 and December 2019. We recruited NSCLC patients in stage I to III. The study’s inclusion and exclusion criteria are displayed in Figure 1. All patients were treated with lobectomy and radical lymphadenectomy. A voluntary tobacco cessation program was offered postoperatively. The program was based on a nationwide initiative of the Federal Centre for Health Education and the Institute for Therapy Research. As in the publication by Park et al. [10], the patients were able to start the program during their primary stay in hospital and thus only a few days after the operation. Patients were taught proven methods of behavioral therapy. Professional psychological support was provided in group and individual sessions. Additionally, patients were allowed to use nicotine replacement therapy such as patches or gum upon individual requests. We defined smoking cessation success as being smoke-free for 12 months after the operation. The evaluation of postoperative smoking behavior was based on voluntary patient reports one year after the operation.

### 2.2. Formation of Study Groups

We formed two study groups from the patient collective that attended re-assessment one year after operation: group A, patients who achieved absolute abstinence from smoking postoperatively; group B, patients who continued smoking postoperatively. The attendance for re-assessment was approximately two-fold higher in group A than that in group B. Regular randomization was not possible at the beginning of the study since we could not predict which patients would achieve definite tobacco abstinence. With regard to reducing bias and imitating randomization, we executed 2:1 matching and formed pairs of patients who were as identical as possible (Figure 1). The pairing was based on age, gender, preoperative smoking habits and UICC stage of lung cancer. At first, we formed pairs of patients in group A. Then, we strictly used the ‘nearest neighbor protocol’ to match a patient from group B to the matched pair of patients from group A.

### 2.3. Data Collection

Patient data were acquired from the hospital’s electronic information system. Patients presented to the out-patient department one year after the operation for a routine surgical check-up on a voluntary basis. A pulmonary function test was performed, and we measured forced expiratory volume within one second (FEV1 in L), as well as diffusion capacity (DLCO in mmol/min/kPa/L). The one-year postoperative values were compared with those of the pre-operative tests (Delta-FEV1 and Delta-DLCO). 

Focusing on the psychological and health axis, we further estimated quality of life one year after operation using the Short Form 36 health questionnaire (SF-36) [11], which all patients completed independently. Its individual dimensions relate to questions about physical functioning (PF: 10 questions), role limitations due to physical health (RP: 4 questions), role limitations due to emotional problems (RE: 3 questions), vitality (VT: 4 questions), mental health (MH: 5 questions), social functioning (SF: 2 questions), body pain (BP: 2 questions), and general health perception (GH: 6 questions). The maximum point value that can be achieved for the individual dimensions is 100 points, which represents a complete absence of health impairments.

Furthermore, the questionnaire’s eight dimensions were grouped into the physical component score (PCS) and the mental component score (MCS) [12]. Both scores were calculated using country-specific weights that are representative of the population in this study [13,14]. These global scores provide information on the patient’s quality of life summarized in only two values. According to Ware et al., both component scores demonstrated good discriminant validity for identifying differences between clinically relevant groups [12].

### 2.4. Statistical Analysis

Pearson’s product–moment correlation, a t-test for independent samples, an analysis of normal distribution (Shapiro–Wilk test) and an analysis of the homogeneity of variance (Levene test) were carried out on a specially designed abacus. Patients’ long-term survival was compared using Kaplan–Meier curves (IBM Corp., Armonk, NY, USA, Released 2017. IBM SPSS Statistics for Windows, Version 25.0. Armonk, NY, USA: IBM Corp.). A *p*-value < 0.05 was considered statistically significant throughout the study.

## 3. Results

### 3.1. Patient Characteristics

In total, 641 patients were surgically treated during the recruitment period. At the time of surgery, 70% (*n* = 449) of the patients were active smokers. Briefly, 62% of active smokers (*n* = 278) successfully quit tobacco use postoperatively, and 171 patients (38%) continued smoking. In total, 124 patients (45%) who quit smoking attended the re-assessment one year after surgery. Only 24% (*n* = 41) of smokers were willing to be re-examined one year after the operation. Group A (smoke-free) comprised 60 patients. In total, 30 patients who continued smoking postoperatively formed the control group (group B) (Figure 1). 

The mean age of the patients was 62.6 ± 12.5 years in group A and 64.3 ± 9.7 years in group B (*p* = 0.82). The gender distribution of the patients was similar in both groups (group A: 64% male; group B: 62%; *p* = 0.46). The preoperative smoking habits measured in pack years were identical in both groups (group A: 47 ± 31; group B: 49 ± 27; *p* = 0.87). Approximately four segments were resected on average in each patient, which was similar in both groups (group A: 4.1 ± 0.4 segments; group B: 4.0 ± 0.4 segments; *p* = 0.91). The patient groups were comparable with regard to UICC stage. (Table 1).

### 3.2. Lung Function and Cessation of Smoking after Lobectomy

**Figure 2:**
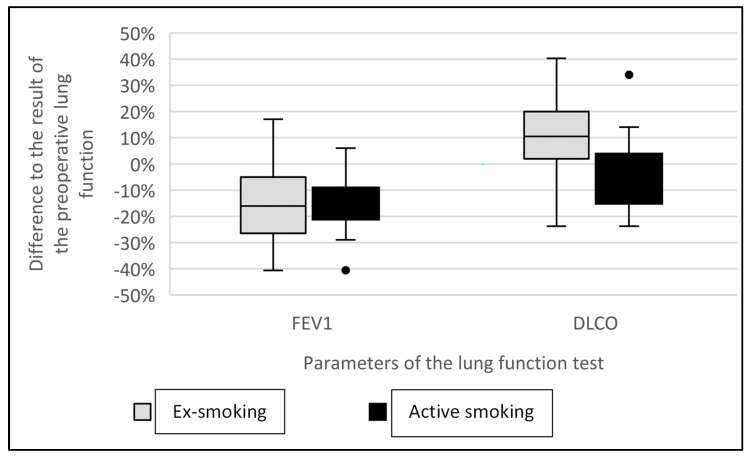
Comparison of pre- and post-operative lung function depending on post-operative smoking behavior. Comparison of the difference compared with the result of preoperative FEV1 (group A: −15 ± 16%; group B: −15 ± 12%; *p* = 0.98) and DLCO (group A: 11 ± 16%; group B: −5 ± 14%; *p* < 0.001) between absolute tobacco abstinence and continued tobacco smoke inhalation after the operation.

**Figure 3:**
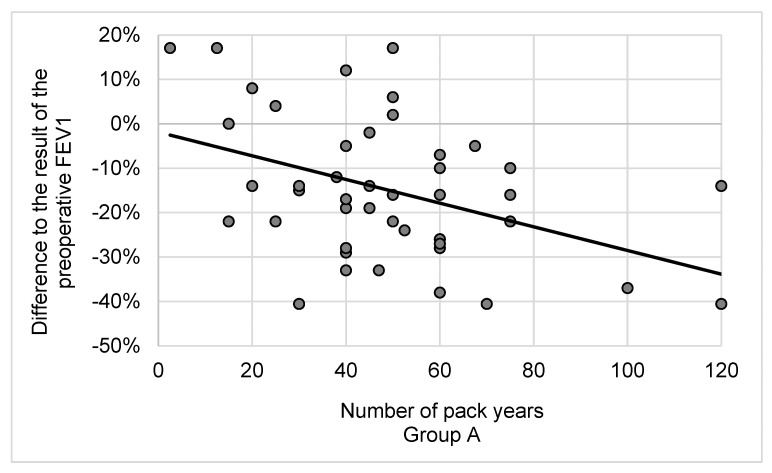

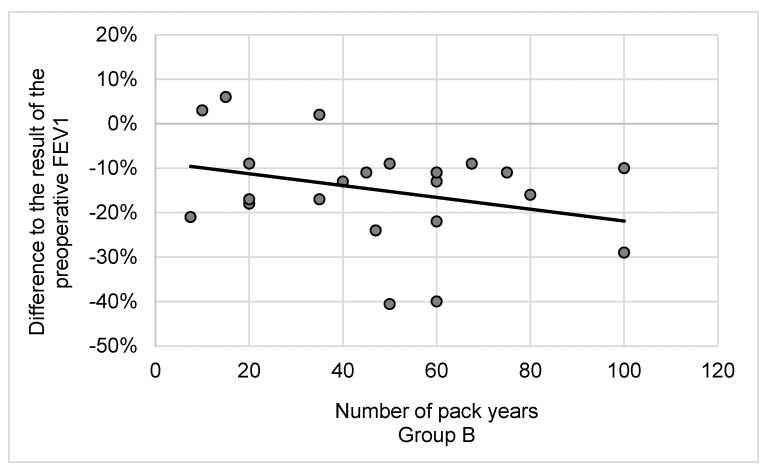
Influence of preoperative cigarette consumption on postoperative FEV1. Correlation between pack years and change in FEV1 after the operation (group A: r = −0.418; *p* = 0.004; group B: r = −0.303; *p* = 0.17). One year after the operation, smokers who successfully ceased smoking had an 11% improvement in DLCO compared to the preoperative values. The DLCO of smokers who continued one year after surgery was 5% worse than that preoperatively (group A: 11 ± 16%; group B: −5 ± 14%; *p* < 0.001) (Figure 2).

FEV1 was decreased equally in both groups one year after lobectomy independent of smoking cessation (group A: −15 ± 16%; group B: −15 ± 12%; *p* = 0.98) (Figure 2). However, the number of preoperative pack years correlated negatively with the result of postoperative FEV1 in group A (r = −0.418; *p* = 0.004). Postoperative FEV1 deteriorated significantly as the number of cigarettes smoked before surgery increased. We could not determine this correlation for group B (r = −0.303; *p* = 0.17) (Figure 3).

### 3.3. Quality of Life and Cessation of Smoking after Lobectomy

**Figure 4:**
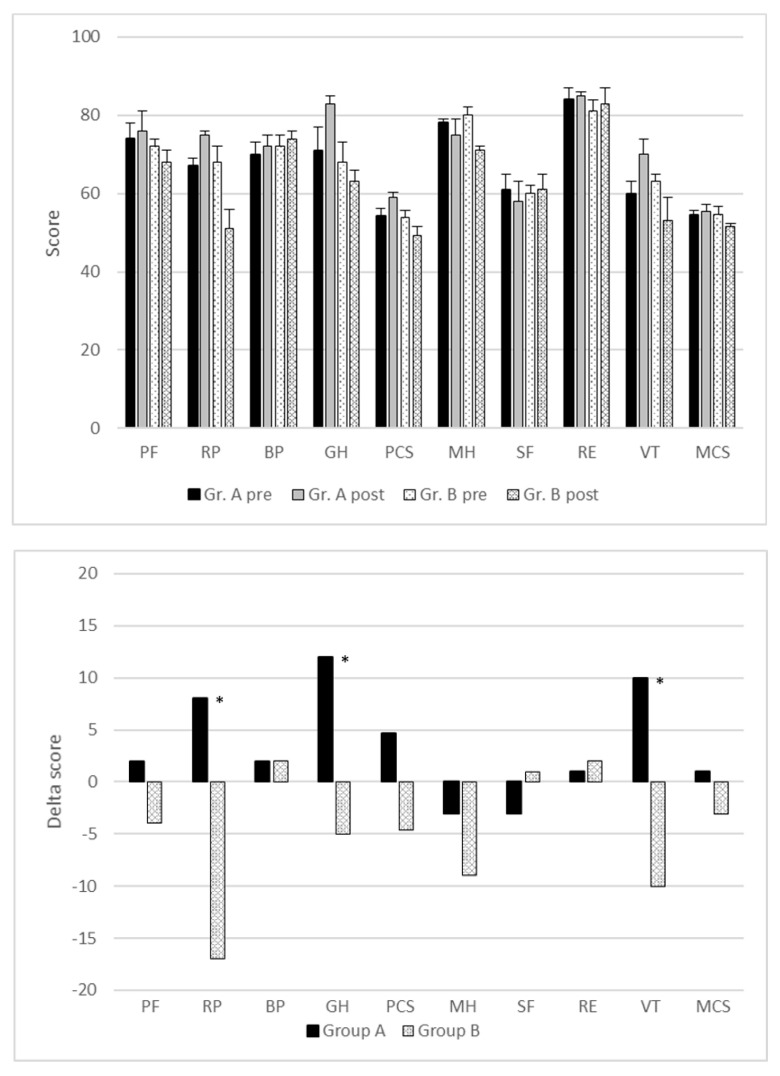
Relationship between postoperative quality of life and tobacco consumption after the operation. Influence of postoperative nicotine abstinence on quality of life one year after lobectomy. (**Above**): All dimensions of the SF-36 questionnaire and the norm-based component scores. (**Below**): Delta scores one year after lobectomy. BP: physical pain; GH: general health perception (*p* = 0.024); MH: mental health; PF: physical health; RE: emotionally related role function, RP: physically conditioned role function (*p* = 0.012); SF: social functioning; VT: vitality (*p* = 0.017). “*” stands for result being “significant”.

The quality of life of the postoperative non-smokers compared to that of the smoking control group was significantly improved one year after the operation in three of eight dimensions of the SF-36 questionnaire. The first dimension that was superior was vitality (VT). Patients in group A scored 10 points higher after tobacco cessation (preoperative: 60 vs. postoperative: 70). The scoring in group B was 10 points lower (63 vs. 53; *p* = 0.017). Significant results were also shown for physical role function (RP) (group A: 67 vs. 75; group B: 68 vs. 51; *p* = 0.012) and for general health perception (GH) (group A: 71 vs. 83; group B: 68 vs. 63; *p* = 0.024). We saw no differences in the remaining five SF-36 dimensions (Figure 4). The delta PCS in group A was +4.6 and that in group B was −3.4 (*p* = 0.078). Furthermore, the delta MCS also showed no significant differences between the two groups (group A: +0.9; group B: −3.1; *p* = 0.29) (Figure 4).

### 3.4. Long-Term Survival and Cessation of Smoking after Lobectomy

**Figure 5:**
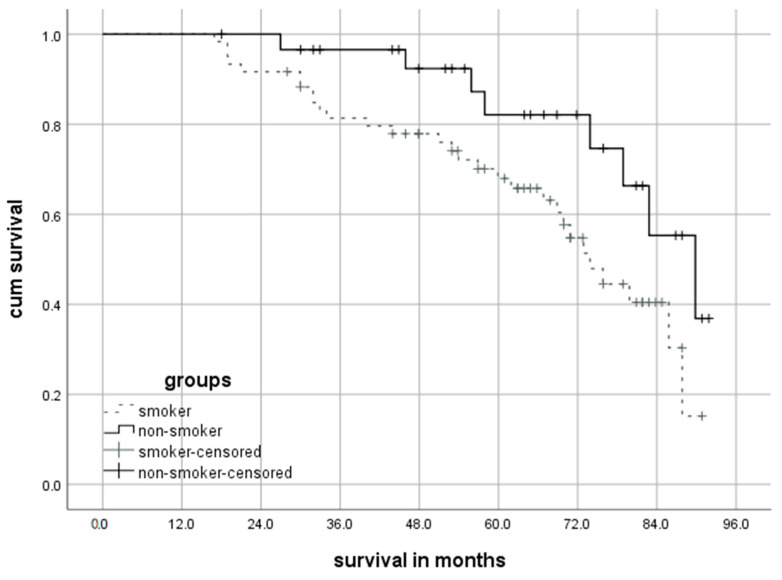
Long-term survival independent of postoperative smoking behavior (Kaplan–Meier curve).

The median survival in months for patients who abstained from smoking after surgery was significantly longer (89.8 ± 6.8 [95%-CI: 76.6–103.1]) compared to that for patients who continued to smoke (73.9 ± 3.6 [95%-CI: 66.9–80.9]; *p* = 0.034). Briefly, 3-year (Group A: 96.2% vs. Group B: 81.0%; *p* = 0.02) and 5-year survival rates (Group A: 80.0% vs. Group B: 64.0%; *p* = 0.016) were significantly higher for patients that stopped smoking after surgery. The hazard ratio (HR) was 2.31 [95%-CI: 1.04–5.13] for postoperative smoking versus tobacco cessation after surgery. Figure 5 shows the results of the long-term survival of the two study groups using the Kaplan–Meier curve.

## 4. Discussion

### 4.1. New Potential for Non-Smokers

To the best of our knowledge, this pioneering study on a homogeneous patient collective formed by pair matching represents the first endeavor to concurrently assess the outcomes of improved lung function, enhanced quality of life, and prolonged survival in postoperative non-smoking after lobectomy for NSCLC, thereby offering a unique and comprehensive perspective on the synergistic effects of smoking cessation interventions. This holistic approach provides invaluable insights into the combined benefits of smoking cessation interventions, informing a more nuanced understanding of their collective influence on patient outcomes and emphasizing the critical role of comprehensive strategies in optimizing postoperative care for NSCLC patients.

The results of our study have the potential to increase the importance of non-smoking programs in the treatment of lung cancer patients. Based on our data, which focus on postoperative smoking cessation, we recommend that patients should be included in a non-smoking program at the latest in the acute hospital and thus a few days after a curative lobectomy. Nevertheless, we emphasize the high relevance of the ERAS guideline, which clearly recommends smoking cessation prior to surgery [15].

Successful completion of a non-smoking program for the patient often requires close cooperation between the family doctor, pulmonologist, oncologist and surgeon. Positive results related to improved lung function, quality of life and long-term survival could provide motivation to the patient.

### 4.2. Lung Cancer Surgery, the Cessation of Smoking and Pulmonary Function

Many patients who actively smoke at the time of lung cancer diagnosis react with an impulse to quit smoking. Currently, there are no clear recommendations for or against tobacco abstinence immediately before or after lobectomy [9]. 

In our study, we show that FEV1 was reduced in both groups one year after the lobectomy irrespective of smoking status. This finding is similar to that of Groth et al., who analyzed the lung function of patients with lung cancer twelve months after a pulmonary resection and showed reduced FEV1 regardless of the smoking status [16]. However, we were able to demonstrate a significantly negative influence of preoperatively accumulated pack years on postoperative FEV1. In contrast, abstinence from tobacco resulted in an 11% improvement in DLCO, whereas the DLCO of continuing smokers was further reduced by 5%. It is known that the presence of CO-hemoglobin hinders the absorption of carbon monoxide in pulmonary function assessments among smokers. Consequently, the measurements may exhibit inferior outcomes in smokers compared to those of non-smokers postoperatively, irrespective of the diffusion distance. Nevertheless, any bias due to different volumes of resected lung tissue can be excluded, since we resected approximately four segments on each patient in both groups.

Rapicetta et al. studied the long-term effects of lobectomy on lung function in lung cancer patients. The mean reduction in FEV1 one year after surgery was 11% [17]. The authors report a significant difference in lung function depending on the preoperative FEV1. One year after the operation, patients with a preoperative FEV1 ≤ 80% had better lung function than did patients in a control group with preoperative FEV1 > 80% (−8% versus −15%; *p* = 0.17) [17].

In a multivariate analysis, Fukui et al. showed that preoperative smoking is a significant predictor (*p* = 0.017) for pulmonary complications after a lobectomy with an odds ratio (OR) of 2.8. The authors also show that a reduction in the preoperative lung function parameters FEV1 (OR 2.6; *p* = 0.001) and DLCO (OR 4.2; *p* = <0.001) predict pulmonary morbidity [18]. In a multivariate analysis, Agostini et al. showed that active smoking at the time of video-assisted lobectomy for NSCLC is the only independent risk factor for postoperative pulmonary complications [19].

According to Lugg et al., active smokers have higher postoperative pulmonary morbidity than do non-smokers (22% versus 2%, *p* = 0.004), a higher need for intensive care therapy (14% versus 0%; *p* = 0.001) and a longer hospital stay (6 versus 5 days, *p* = 0.001). The authors observed a trend for a better postoperative course in patients who stopped tobacco consumption more than six weeks before the surgery [20]. In contrast, Rodriguez et al. showed that initiating nicotine cessation immediately before lobectomy for NSCLC does not lead to altered postoperative pulmonary complications and suggest that postponing an operation depending on smoking status is not justified [21]. 

### 4.3. Influencing Quality of Life through Tobacco Cessation

In 2019, Martinez et al. examined the quality of life of patients with different tumor types, including lung cancer. The study showed a longitudinal relationship between quality of life and the cessation of tobacco smoke inhalation. The longer the abstinence, the better the quality of life [22]. Andreas et al. summarized the positive effects of postoperative nicotine abstinence in patients with NSCLC in a review article. The authors made particular reference to the improved general health of postoperative non-smokers [23]. 

Furthermore, Bloom et al. showed that abstinence from smoking after surgery for lung cancer was associated with fewer depressive symptoms and less fatigue [24]. In their prospective study, Danson et al. examined the quality of life of patients with advanced lung cancer. Patients who did not stop smoking after the diagnosis reported a poorer quality of life than did ex-smokers. In this study, smoking status was a significant independent predictor of cough (patients with T1 tumors) and poorer cognitive function (T2 tumors) [25]. 

According to Levy et al. quality of life is already improved after one month of tobacco abstinence. Ex-smokers six months after tobacco cessation report on good health 30% more often and on psychological stress 19% less often [3].

In our study, stopping tobacco consumption significantly improved vitality, physical role function and general health perception one year after surgery. In contrast, ceasing smoking did not lead to an effect on role limitations due to physical health or emotional problems, mental health, social functioning or body pain. Furthermore, both global scores (PCS and MCS) were also not significantly influenced by the postoperative cessation of tobacco consumption.

### 4.4. Smoking Behavior Affects Long-Term Survival

In our study, we were able to demonstrate a significant 3-year and 5-year survival benefit for patients who quit smoking postoperatively, which also translates to longer median survival. The reasons for this survival benefit appear to be multifactorial. As early as 2009, Liptay et al. showed that good DLCO is a significant prognostic factor for long-term survival after lobectomy [26]. Consequently, improved DLCO in postoperative non-smokers may also be a factor in our study for the survival benefit. 

In non-smokers, a well-differentiated pulmonary adenocarcinoma, which is associated with a better prognosis, is more likely to develop [26]. Ozeki et al. examined the connection between DLCO and the histopathology of pulmonary adenocarcinomas. The authors concluded that pulmonary damage is not only associated with carcinogenesis but also with tumor progression [27].

Parsons et al. showed significantly reduced long-term survival in patients with NSCLC who did not quit smoking after surgery. In these patients, both the risk of developing secondary tumors (2.3-fold) and the risk of recurrence (1.9-fold) were increased. The overall mortality in postoperative smokers was 2.9 times higher than that in non-smokers [28]. The authors hypothesized that the better long-term survival achieved after smoking cessation is due to reduced tumor progression rather than due to a reduction in cardio-respiratory deaths [28]. 

According to a review by Florou et al., continued smoking leads to significantly increased overall mortality [29]. As reasons for this development, the authors postulate not only an increased incidence of side effects during cancer treatment but also a poorer response to chemotherapy. This is based on nicotine-induced resistance and the modulation of mitochondrial signal transmission. Even the success of targeted cancer therapies such as Erlotinib is reduced by nicotine consumption [29]. Continued smoking appears to result in not only increased toxicity, but also the reduced effectiveness of cancer therapy. This is directly associated with lower response rates, especially in patients with advanced disease [6]. In line with these conclusions, Andreas et al. postulate an improved response to chemotherapy through smoking cessation [22].

### 4.5. Study Limitations

Our tobacco cessation program was attended on a voluntary basis and there was no obligation of the patients to attend the re-assessment one year after surgery. An objective control method such as urine cotinine or inhaled CO was not implemented to assess the smoking status of the patients. The willingness of patients to attend our follow-up was approximately two-fold higher in group A than that in group B. After completing the re-assessment, we had a skewed sample that was small in terms of the number of complete data sets. Furthermore, the sample size, while sufficient for our primary analysis, may limit the generalizability of our results. Lastly, caution should be exercised in extending our findings to broader lung cancer populations, warranting further investigations for a more comprehensive understanding.

A critical imperative lies in conducting additional large-scale multicenter studies on smoking cessation. These investigations are essential for a rigorous evaluation of interventions, encompassing diverse populations, to refine strategies and advance our scientific understanding of tobacco dependence cessation.

## 5. Conclusions

The positive long-term effects of postoperative smoking cessation should be known to all physicians involved in the treatment of lung cancer. Hoping to achieve a better quality of life, improved lung function and prolonged survival, lung cancer patients might be motivated to stop active tobacco consumption. It is of paramount importance for the treating physicians to use the time of diagnosis as a “window of opportunity” and support their lung cancer patients to quit smoking.

## Figures and Tables

**Table 1:** Patient characteristics.

	Preoperative Smokers (*n* = 449)		Group A (*n* = 60)		Group B (*n* = 30)		SMD	*p*-Value
*Demographic data*								
Age (Years)	63.7 ± 11.3		62.6 ± 12.5		64.3 ± 9.7		0.14	0.82
Male (%)	65		64		62			0.46
*Comorbidity*								
COPD (%)	35		38		29			0.31
BMI (kg/m²)	26.1 ± 5.2		27.1 ± 5.3		23.7 ± 3.9		0.26	0.11
Prev. oncol. disease (%)	4		3		5			0.18
*Smoking habit*								
Pack years	45 ± 34		47 ± 31		49 ± 27		0.04	0.87
*Lobectomy*								
RUL (%)	26		25		28			
ML (%)	5		5		7			
RLL (%)	19		22		16			0.21
LUL (%)	26		23		28			
LLL (%)	24		25		21			
Mean numb. of res. seg.	4.0 ± 0.5		4.1 ± 0.4		4.0 ± 0.4		0.2	0.91
*Histologic subtype*								
Adenocarcinoma (%)	46		52		46			
Squ. cell carcinoma (%)	41		38		39			0.23
Large cell carcinoma (%)	9		8		11			
Others (%)	4		2		4			
*NSCLC stage*								
	*n*	%	*n*	%	*n*	%		
IA1	72	16	8	13	5	17		
IA2	22	5	7	12	2	7		
IA3	41	9	5	8	3	10		
IB	63	14	7	12	4	13		0.41
IIA	81	18	11	18	5	17		
IIB	85	19	11	18	6	20		
IIIA	67	15	10	17	4	13		
>IIIA	18	4	1	2	1	3		

BMI: body mass index; COPD: chronic obstructive pulmonary disease; LLL: left lower lobe; LUL: left upper lobe; Mean numb. of res. seg.: mean number of resected segments; ML: middle lobe; Prev. oncol. disease: previous oncological disease; RLL: right lower lobe; RUL: right upper lobe; Squ. cell carcinoma: squamous cell carcinoma.

## Data Availability

The data presented in this study are available on request from the corresponding author.
